# Telemedicine Improves Performance of a Two-Incision Lower Leg Fasciotomy by Combat Medics: A Randomized Controlled Trial

**DOI:** 10.1093/milmed/usad486

**Published:** 2023-12-22

**Authors:** Pieter W Stark, Boudewijn L S Borger van der Burg, Thijs T C F van Dongen, Marnalg Casper, 1 Wouter, Rigo Hoencamp

**Affiliations:** Trauma Research Unit, Department of Surgery, Erasmus MC University Hospital, Rotterdam, South Holland 3015 GD, The Netherlands; Department of Surgery, Alrijne Hospital, Leiderdorp, South Holland 2353 GA, The Netherlands; Department of Surgery, Alrijne Hospital, Leiderdorp, South Holland 2353 GA, The Netherlands; Department of Surgery, Alrijne Hospital, Leiderdorp, South Holland 2353 GA, The Netherlands; Ministry of Defense, Defense Healthcare Organization, Den Haag, South Holland 2511 CB, The Netherlands; Ministry of Defense, Defense Healthcare Organization, Den Haag, South Holland 2511 CB, The Netherlands; Ministry of Defense, Defense Healthcare Organization, Den Haag, South Holland 2511 CB, The Netherlands; Trauma Research Unit, Department of Surgery, Erasmus MC University Hospital, Rotterdam, South Holland 3015 GD, The Netherlands; Department of Surgery, Alrijne Hospital, Leiderdorp, South Holland 2353 GA, The Netherlands; Ministry of Defense, Defense Healthcare Organization, Den Haag, South Holland 2511 CB, The Netherlands; Department of Surgery, Leiden University MC, Leiden, South Holland 2333 ZA, The Netherlands

## Abstract

**Introduction:**

The primary aim of this randomized controlled trial was to assess if a head-mounted display (HMD) providing telemedicine support improves performance of a two-incision lower leg fasciotomy by a NATO special operations combat medic (combat medic).

**Materials and Methods:**

Thirty-six combat medics were randomized into two groups: One group performed a two-incision lower leg fasciotomy with the assistance of an HMD, while the control group completed the procedure without guidance. A Mann–Whitney U test was used to determine the possible differences in release of compartments and performance scores, as assessed by a supervising medical specialist. A Fisher’s exact test was used to compare the proportions of collateral damage between groups. An independent-samples *t*-test was used to interpret total procedure times. The usability and technical factors involving HMD utilization were also assessed.

**Results:**

Combat medics in the HMD group released the anterior compartment (*P* ≤ .001) and deep posterior compartment (*P *= .008) significantly better. There was significantly more iatrogenic muscle (*P *≤ .001) and venous damage (*P *≤ .001) in the control group. The overall performance of combat medics in the HMD group was significantly better than that of the control group (*P* < .001). Combat medics in the control group were significantly faster (*P *= .012). The combat medics were very satisfied with the HMD. The HMD showed no major technical errors.

**Conclusions:**

This randomized controlled trial shows that a HMD providing telemedicine support leads to significantly better performance of a two-incision lower leg fasciotomy by a combat medic with less iatrogenic muscle and venous damage.

## INTRODUCTION

In armed conflicts, because of the nature of their work and the hostile environment, military personnel are at high risk for traumatic injuries. The tactical combat casualty care guidelines were developed to provide guidance for medical care on the battlefield and in austere environments.^1^ Tactical combat casualty care is divided into care under fire, tactical field care, and tactical evacuation care.^2^ The mechanism and type of injury and the tactical situation determine in which phase of tactical combat casualty care certain medical procedures can be performed.^2^ Life- and limb-saving procedures are usually performed by forward surgical teams or by experienced medical specialists in medical treatment facilities.^3^

If no forward surgical teams are available and evacuation to a medical treatment facility is impossible, the principles of prolonged field care are applied. Prolonged field care is defined by NATO as field medical care, beyond “doctrinal planning time-lines” by a NATO special operations combat medic in order to decrease patient mortality and morbidity. When the term combat medic is used in the remainder of this article, it refers to a NATO special operations combat medic. Prolonged field care utilizes limited resources and is sustained until wounded military personnel arrive at the next appropriate level of care.^4^ Increased treatment capability and reduced prehospital transport times are likely contributors of casualty survival during the Afghanistan conflict despite evidence of increased severity and complexity of wounds.^5^ Recent conflicts, such as the war in Ukraine, have shown greater complexity, which decreases the availability of medical care and prolonged evacuation time.^6^ In these conflicts, prolonged field care has become increasingly more important.

In battle casualties from NATO coalition forces in Iraq and Afghanistan, the extremities were affected in 39% of cases, compared to head and neck (31%), truncal (27%), and other injuries (3%).^7^ Penetrating injuries, such as gunshot wounds, crush injuries, vascular injuries, and fractures of the extremities, increase the risk of developing compartment syndrome.^8^ Timely performance of a two-incision lower leg fasciotomy is an example of a life- and limb-saving procedure, which is performed when compartment syndrome is suspected after acute traumatic injury. Under specific conditions, for example, with appropriate medical equipment available and under the premise of adequate training, parts of life- and limb-saving procedures can also be performed by a combat medic when no experienced medical specialist is available on site. This might be essential, as prolonged field care without access to a medical treatment facility may take several days and an incomplete or delayed two-incision lower leg fasciotomy is associated with nerve and/or vascular damage and muscle necrosis, which can lead to loss of function, major amputation, and mortality.^9^

Proficiency in performing a two-incision lower leg fasciotomy can be difficult to acquire and retain, even for practicing medical specialists.^10^ Depending on the scope and depth of training provided to combat medics, telemedicine might be the preferred option to safely perform life- and limb-saving surgery procedures during prolonged field care. The information-sharing capacity of telemedicine enables access to a remote medical specialist to support medical decision-making in prolonged field care.^11,12^ A head-mounted display (HMD) is a recently developed option for telemedicine support.^13^ An HMD is a wearable technology that presents data onto lenses and records images or videos through a front-facing camera.^13^ This enables the consulted medical specialist to observe and supervise a surgical intervention from a different location and use telestration for precise instructions. Telestration is defined as a technique for enabling annotations over an image or video.^14^ Our group recently published a feasibility study on the use of an HMD providing telemedicine support for a combat medic during a two-incision lower leg fasciotomy.^15^ Other studies also suggest that an HMD can be successfully utilized to provide telemedicine support for the combat medic when a two-incision lower leg fasciotomy is performed.^12,16^ The sparsely available evidence does not show whether utilization of an HMD significantly improves the performance of a two-incision lower leg fasciotomy by a combat medic compared to non-supervised procedures.

The primary aim of this randomized controlled trial (RCT) was to assess if an HMD providing telemedicine support improves performance of a two-incision lower leg fasciotomy by a combat medic. The secondary aim was to assess the self-perceived usability and technical performance of the HMD by combat medics.

## METHODS

This RCT was conducted in the Skillslab & Simulation Center of Erasmus University Medical Center, Rotterdam, the Netherlands. The AnubiFiX-embalmed post-mortem human specimens^17,18^ were donated for scientific research and medical training at the Anatomy Department of Skillslab, Rotterdam, and were part of a national body donation program approved by Dutch law and regulations. A protocol for this study was reviewed and approved by the Dutch Ministry of Defense and the Institutional Review Board of Alrijne Hospital, the Netherlands (NWMO 17-15, 17.409rt.tk).

### Hardware and Software

**FIGURE 1.**
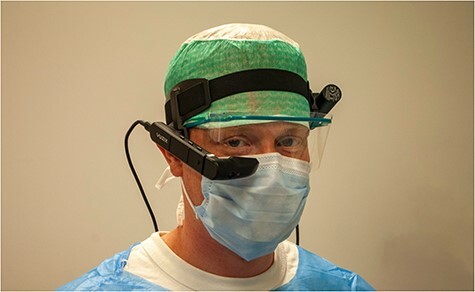
An example of a combat medic wearing the Vuzix M400 head-mounted display.

The Vuzix M400 device (Fig. 1; Vuzix, Rochester, NY, USA) was selected as HMD, based on hardware and software characteristics. A MacBook Air 2017 device (Apple, Cupertino, CA, USA) was used to observe and telestrate the two-incision lower leg fasciotomy. An iPhone 11 2020 device (Apple, Cupertino, CA, USA) was used to establish the connection between the combat medic and the supervising medical specialist by scanning a Quick Response code, which generated a call to the MacBook of the supervising medical specialist. The HMD and MacBook were connected to a WPA-2-encrypted WiFi network. Gemvision software (Gemvision, Rotterdam, the Netherlands) was installed on the MacBook and on the HMD used. Gemvision software is certified with ISO 27001 and NEN 7510 standards for information security in health care.

### Study Procedures

Volunteers from a group of non-surgical-trained NATO special operations combat medics (combat medics) were recruited to participate in this RCT. All combat medics completed an informed consent to participate in this RCT. Two medical specialists with expertise in performing a two-incision lower leg fasciotomy acted as supervisors. All combat medics attended a standard concise formalized lecture. The lecture contained indications for a two-incision lower leg fasciotomy, basic anatomy of the lower leg, and instructions and surgical techniques for performing a two-incision lower leg fasciotomy. The lecture also included details and instructions for the utilization of the HMD. The combat medics were randomized by drawing a numbered card from a bag (one = HMD, zero = control group) into a group using the HMD (HMD group) or into a group performing a two-incision lower leg fasciotomy without guidance from a medical specialist (control group). To make sure both groups contained a sufficient number of participants for analysis, a 1:1 ratio for randomization was chosen. Randomization took place after the standard concise lecture to ensure that combat medics were not prejudiced. Blinding was not possible for both combat medics and supervising medical specialists as the HMD group received remote supervision, while the control group performed a two-incision lower leg fasciotomy without guidance. The study was conducted in 30-minute time blocks, with one combat medic in the HMD group and one combat medic in the control group per time block. All combat medics performed a two-incision lower leg fasciotomy on an AnubiFiX-embalmed post-mortem human leg^17,18^ in an operating room with access to regular surgical instruments. In the HMD group, a medical specialist was located at a separate location without the possibility of direct contact with the combat medics during the performance of the two-incision lower leg fasciotomy.

### Study Parameters

The main study parameter was the opening of the individual compartments of the lower leg by a combat medic. The supervising medical specialist examined the leg in the operating room after the two-incision lower leg fasciotomy was finished to assess if all four compartments were released and to assess possible iatrogenic damage to muscles, nerves, arteries, and veins. The release of the individual compartments was scored as not released, partially released, fully released, or unable to assess.

Iatrogenic damage was a secondary study parameter. It was scored as no damage, muscular damage, nerve damage, arterial damage, venous damage, or unable to assess. The performance of the combat medic was also a secondary study parameter, which was assessed by the supervising medical specialist using the Resident’s Operative Performance Tool (ROPT).^19^ The ROPT, a validated assessment tool, consists of nine items. Each item is scored on a one-to-five scale, with one indicating poor performance, three representing competence, and five indicating high performance. If the consulted surgeon was unable to assess a certain item it was scored with zero. Some items had additional information to support scoring. Additional secondary study parameters included the mean procedure time, recorded in minutes and seconds. The time to set up the HMD and its connection was excluded from the mean procedure time calculation. For both groups, the mean procedure time started when the combat medic initiated the two-incision lower leg fasciotomy by picking up the scalpel. The perceived usability of the HMD was assessed with the Telehealth Usability Questionnaire (TUQ)^20^ by the performing combat medic. TUQ is a validated tool developed to evaluate the usability of telehealth implementation and services. It has twenty-one items across six usability domains: Usefulness (three items), ease of use and learnability (three items), interface quality (four items), interaction quality (four items), reliability (three items), and satisfaction and future use (four items). Each item utilizes a seven-point Likert scale, ranging from 1 (least usable) to 7 (most usable). The technical performance of the HMDs was retrospectively evaluated by the researchers.

### Statistical Analyses

Statistical analyses were performed in collaboration with a statistician expert, using the Statistical Package for the Social Sciences (version 28, 2021, IBM Corporation, Armonk, NY, USA). The median and range of the release were calculated for each compartment of the two-incision lower leg fasciotomy. A Mann–Whitney U test was used to determine differences in compartment release between the HMD group and control group. Distributions of compartment release for both groups were similar as assessed by visual inspection. Therefore, differences between the HMD group and control group were calculated using median (range). G*Power (G*Power, version 3.1.9.6, 2023, Heinrich Heine University, Düsseldorf, Germany)^21^ was used to compute a required sample size based on the Mann–Whitney U test for the main study parameter. Given an α error probability of.05, a power (1 − β error probability) of 0.8, and effect size of 0.5, a total required sample size of 106 was calculated.

The proportions of damage to muscle, nerves, arteries, and veins were calculated for both groups. Because of sample sizes, Fisher’s exact test was used to compare the proportions of damage between the HMD group and control group. The median and range were calculated for each question of the ROPT. To determine differences in performance scores, a Mann–Whitney U test was used. The visual inspection showed that the distribution of performance scores was similar. Therefore, differences between the HMD group and control group were calculated using median (range). An independent-samples *t*-test was used to determine if there were differences in total procedure time between the HMD group and the control group. Total procedure times were normally distributed, as assessed by Shapiro–Wilk’s test (*P* > .05). For each question of the TUQ, the median and range were calculated.

## RESULTS

Thirty-six combat medics participated in this study. None of the combat medics had previously performed a two-incision lower leg fasciotomy. Eighteen combat medics were enrolled in the HMD group, and 18 combat medics were enrolled in the control group.

**FIGURE 2.**
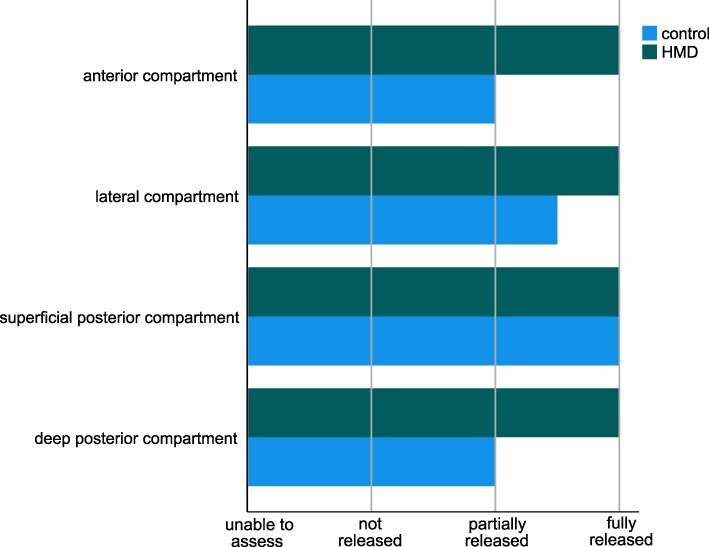
Adequacy of two-incision lower leg fasciotomy (median*).

The anterior compartment and deep posterior compartment were fully released by combat medics in the HMD group, which was significantly better than combat medics in the control group who only partially released these compartments (Fig. 2; anterior compartment: *P* < .001, deep posterior compartment: *P* = .008). The lateral compartment was more often fully released by combat medics in the HMD group, compared to combat medics in the control group although not to a statistically significant degree (Fig. 2; lateral compartment: *P *= .051). The superficial posterior compartment was fully released in both groups (Fig. 2; superficial posterior compartment: *P *= .406).

*Mann–Whitney U test.

**FIGURE 3.**
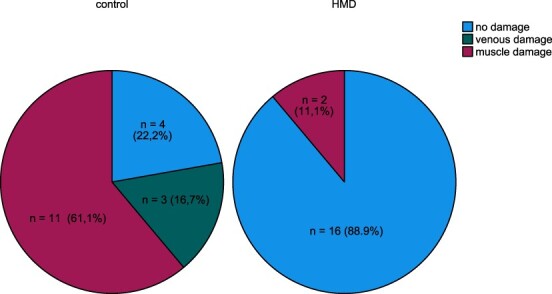
The damage caused by the combat medic (percentages*).

Eleven combat medics (Fig. 3; *n* = 11/18, 61.1%) in the control group caused muscle damage, which is significantly more than the HMD group in which only two combat medics (Fig. 3; *n* = 2/18, 11.1%) caused muscle damage (*P* < .001). Three combat medics (Fig. 3; *n* = 3/18, 16.7%) in the control group caused venous damage, which is significantly more than the HMD group in which none of the combat medics caused venous damage (*P *< .001). In these three cases, the great saphenous vein was damaged. None of the combat medics caused damage to nerves or arteries.

*Fisher’s exact test.

The results of the ROPT showed that the overall performance of combat medics in the HMD group was considered competent, which was significantly better than the control group, who had a very poor performance (HMD group: Median 3, range 2 vs. control group: Median 1, range 2; *U *= 311, *P* < .001).

**FIGURE 4.**
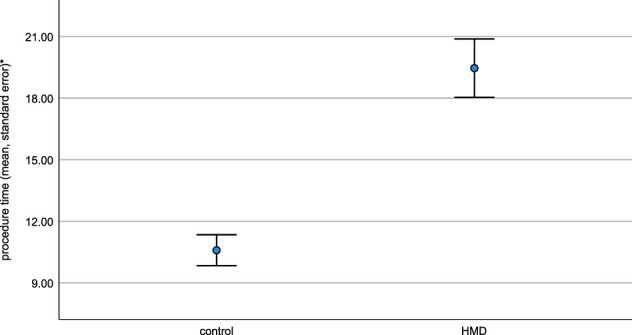
The procedure time in minutes:seconds (mean, standard error*).

The mean procedure time in the HMD group was 19 minutes and 39 seconds (Fig. 4; SE 01:24), and the mean procedure time in the control group was 10 minutes and 50 seconds (Fig. 4; SE 00:45), which made the control group significantly faster (Fig. 4; *P *= .012).

*Independent-samples *t*-test.

As shown in the Supplementary Material (Supplemental 1, range 1–7), the combat medics expressed a high level of satisfaction with the use of HMDs for receiving telemedicine support during the performance of a two-incision lower leg fasciotomy (median 6.5, range 2). There was a strong consensus about using an HMD for future telemedicine support (median 7, range 2). The combat medics reported that utilizing an HMD would improve their access to telemedicine support (median 7, range 3). The use of HMDs was linked to improved self-expression among the combat medics (median 6.5, range 3), which made them feel comfortable communicating with the supervising specialist (median 7, range 2). The supervising medical specialists found it straightforward to communicate with the combat medics through the HMD. The medical specialists were also satisfied with the HMD.

The combat medics utilizing the HMD experienced no poor quality of connection or connection failure. In all cases, the call between the combat medic and the medical specialist was set up in one or two attempts. Setting the correct position of the HMD needed a few minutes in order to allow for adequate telemedicine support. After the HMD was positioned correctly, the image quality was sufficient. In one case, the call was disconnected by accident, which was corrected quickly. There were no incidents where the call failed completely. No reports were made about adverse effects from wearing the HMD.

## DISCUSSION

This RCT shows that an HMD providing telemedicine support significantly improves opening of the individual compartments of the lower leg by a combat medic with less iatrogenic muscle and venous damage. The HMD was used with great satisfaction by the combat medics.

As casualty care is changing in current conflicts with a higher emphasis on longer evacuation times, life- and limb-saving procedures will be performed in more remote areas. Telemedicine might be useful as an adjunct to provide remote support from a medical specialist for the combat medic when a life- or limb-saving procedure is performed during prolonged field care.^22^ Previous studies showed the feasibility of an HMD providing telemedicine support for military medical personnel during the performance of a two-incision lower leg fasciotomy.^12,15,16^ Other studies show the feasibility of an HMD providing telemedicine support for a two-incision lower leg fasciotomy in austere environments but for non-military medical personnel.^23,24^ The accurate opening of the compartments, the minimal damage to muscles and veins, and the ROPT results clearly show that an HMD providing telemedicine support leads to significantly better performance of a two-incision lower leg fasciotomy by a combat medic. An interesting observation from the results is that combat medics in the control group also adequately released one compartment, a result that the supervisors attributed to the effect of prior training. These findings indicate that telemedicine support is a valuable adjunct for the combat medic when performing life- and limb-saving procedures while forward surgical teams are not available and timely evacuation to a medical treatment facility is not possible.

Combat medics in the control group performed the two-incision lower leg fasciotomy significantly faster, but they more often caused damage to great saphenous vein and muscles. The goal of a two-incision lower leg fasciotomy is not to perform the procedure as fast as possible but to adequately open the individual compartments of the lower leg. Combat medics in the HMD group used the HMD to adequately release all compartments and to prevent collateral damage. Currently, legislation does not allow combat medics to perform complex surgical life- and limb-saving procedures, which, in a civilian setting, are only performed by surgeons or surgical residents. Utilizing an HMD providing telemedicine support might accommodate the required supervision from a medical specialist allowing combat medics to provide these life- and limb-saving procedures. There were no major technical errors that hindered telemedicine support during the performance of a two-incision lower leg fasciotomy in a controlled environment. Future research should focus on the added value of an HMD in a real austere environment and tests with different options for connectivity. In such a study set-up, the full potential of the HMD and options for data security could be tested with current military means of communication, as this might reflect the military setting more adequately.

This RCT has some limitations. Fewer combat medics than required by the computed sample size were included. In the Netherlands, a limited number of combat medics are available to participate in research because of operational tasking. In addition, the number of non-surgical trained combat medics is limited. An AnubiFiX-embalmed post-mortem human leg^17,18^ was used for the two-incision lower leg fasciotomy. Although it does not perfectly replicate acute compartment syndrome, it remains the most realistic model currently available for practicing a two-incision lower leg fasciotomy. This RCT took place within a controlled training operation room with access to regular surgical instruments, which differs from austere environments because of various external factors. Future research should focus on the use of HMDs in (simulated) austere environments including the available surgical set as used by combat medics during prolonged field care. It is also interesting to include other procedures in future research, such as cricothyrotomy, wound debridement, and regional pain blocks, to investigate the potential impact of an HMD on the performance of the combat medic. Besides the tactical situation in austere environments, the urgency of acute traumatic injuries determines options to establish connection between a combat medic with an HMD and remote medical specialists. Knowledge about and adequate training in life- and limb-saving procedures remain essential for the combat medic before considering the HMD.

## CONCLUSION

This RCT shows that an HMD providing telemedicine support leads to significantly better performance of a two-incision lower leg fasciotomy by a combat medic with less iatrogenic muscle and venous damage. The outcome of this study indicates that telemedicine support is a valuable adjunct for the combat medic when performing life- and limb-saving procedures in austere environments.

## Supplementary Material

usad486_Supp

## Data Availability

The data underlying this article will be shared on reasonable request to the corresponding author. All data are freely accessible.
