# Supplementary figures and images for: Telemedicine Improves Performance of a Two-Incision Lower Leg Fasciotomy by Combat Medics: A Randomized Controlled Trial
Source: Mil Med. 2023 Dec 22;189(7-8):e1668–74. doi: 10.1093/milmed/usad486 (PMC11221554; doi:10.1093/milmed/usad486)

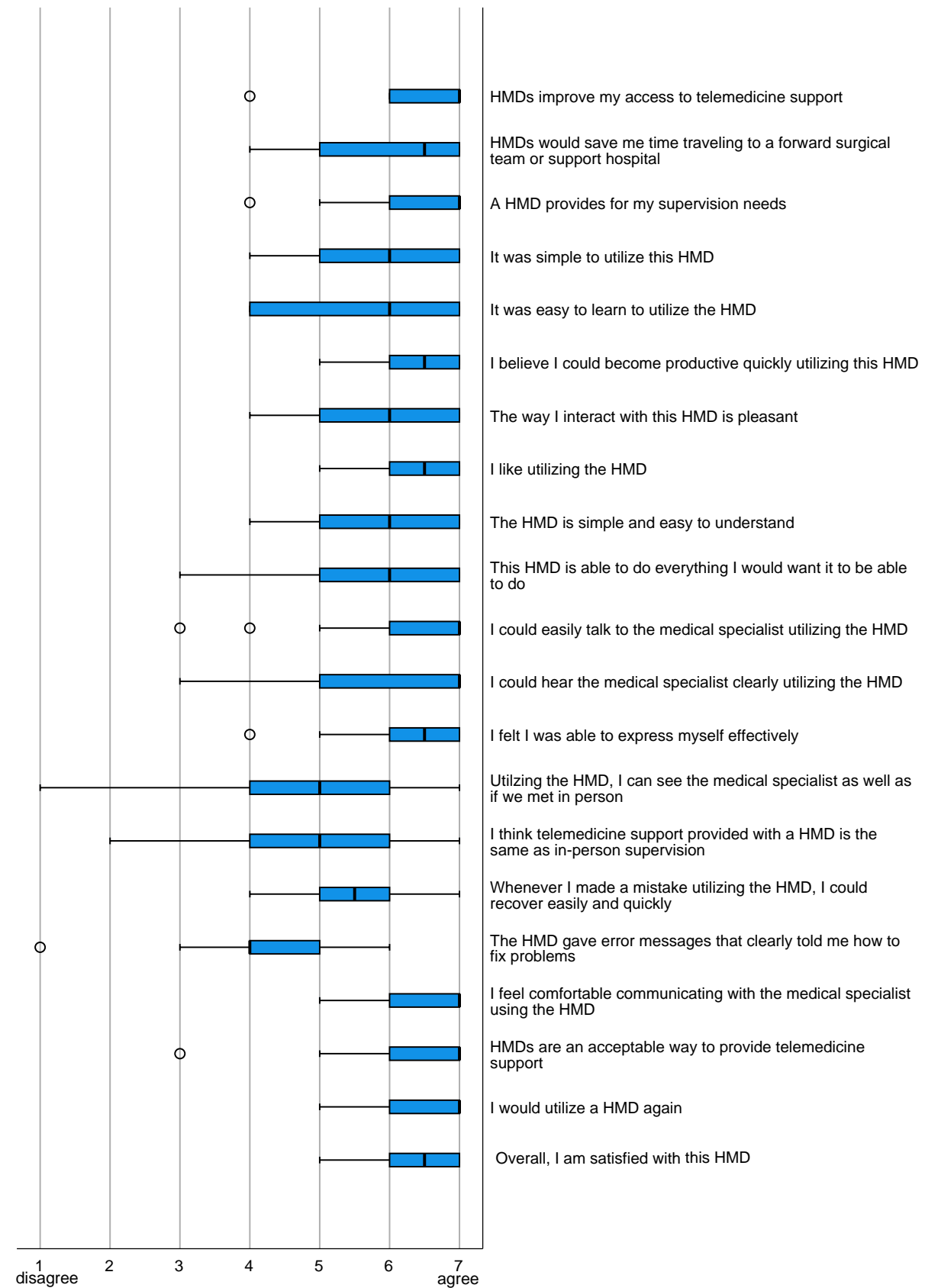

Supplemental 1: Usability scores (median, IQR\*)

\*Mann Whitney U test

Supplement: usad486_Supp [file usad486_supp.zip › Supplemental 1 version 4.2 10032023.pdf]
